# Diagnostic MicroRNA Signatures to Support Classification of Pulmonary Hypertension

**DOI:** 10.1161/CIRCGEN.124.004862

**Published:** 2025-04-18

**Authors:** Niamh Errington, Li Zhou, Christopher J. Rhodes, Yiu-Lian Fong, Lihan Zhou, Sokratis Kariotis, Eileen Harder, Aaron Waxman, Timothy Jatkoe, John Wharton, A.A. Roger Thompson, Robin A. Condliffe, David G. Kiely, Luke S. Howard, Mark Toshner, Cheng He, Dennis Wang, Martin R. Wilkins, Allan Lawrie

**Affiliations:** 1 National Heart and Lung Institute, Imperial College London, United Kingdom (N.E., C.J.R., J.W., L.S.H., D.W., M.R.W., A.L.).; 2 MiRXES Lab, Singapore, Republic of Singapore (Li Zhou, Lihan Zhou, C.H.).; 3 Janssen Pharmaceutical Companies of Johnson & Johnson, Raritan, NJ (Y.-L.F., T.J.).; 4 Bioinformatics Institute, Agency for Science, Technology and Research (A*STAR), Singapore, Republic of Singapore (S.K., D.W.).; 5 Brigham and Women’s Hospital and Harvard Medical School, Boston, MA (E.H., A.W.).; 6 Division of Clinical Medicine, The University of Sheffield, United Kingdom (A.A.R.T., R.C., D.G.K.).; 7 Sheffield Pulmonary Vascular Disease Unit, Sheffield Teaching Hospitals Foundation Trust and NIHR BRC Sheffield, United Kingdom (A.A.R.T., R.C., D.G.K.).; 8 Hammersmith Hospital, Imperial College Healthcare NHS Trust, London, United Kingdom (L.S.H.).; 9 Papworth NHS Foundation, Cambridge, United Kingdom (M.T.).; 10 Department of Medicine, University of Cambridge, United Kingdom (M.T.).

**Keywords:** biomarkers, early diagnosis, machine learning, miRNA, pulmonary hypertension

## Abstract

**BACKGROUND:**

Patients with pulmonary hypertension (PH) are classified based on disease pathogenesis and hemodynamic drivers. Classification informs treatment. The heart failure biomarker NT-proBNP (N-terminal pro-B-type natriuretic peptide) is used to help inform risk but is not specific to PH or sub-classification groups. There are currently no other biomarkers in clinical use to help guide diagnosis or risk.

**METHODS:**

We profiled a retrospective cohort of 1150 patients from 3 expert centers with PH and 334 non-PH symptomatic controls (disease controls) from the United Kingdom to measure circulating levels of 650 microRNAs (miRNAs) in serum. NT-proBNP (ELISA) and 326 well-detected miRNAs (polymerase chain reaction) were prioritized by feature selection using multiple machine learning models. From the selected miRNAs, generalized linear models were used to describe miRNA signatures to differentiate PH and pulmonary arterial hypertension from the disease controls, and pulmonary arterial hypertension, PH due to left heart disease, PH due to lung disease, and chronic thromboembolic pulmonary hypertension from other forms of PH. These signatures were validated on a UK test cohort and independently validated in the prospective CIPHER study (A Prospective, Multicenter, Noninterventional Study for the Identification of Biomarker Signatures for the Early Detection of Pulmonary Hypertension) comprising 349 patients with PH and 93 disease controls.

**RESULTS:**

NT-proBNP achieved a balanced accuracy of 0.74 and 0.75 at identifying PH and pulmonary arterial hypertension from disease controls with a threshold of 254 and 362 pg/mL, respectively but was unable to sub-categorize PH subgroups. In the UK cohort, miRNA signatures performed similarly to NT-proBNP in distinguishing PH (area under the curve of 0.7 versus 0.78), and pulmonary arterial hypertension (area under the curve of 0.73 versus 0.79) from disease controls. MicroRNA signatures outperformed NT-proBNP in distinguishing PH classification groups. External testing in the CIPHER cohort demonstrated that miRNA signatures, in conjunction with NT-proBNP, age, and sex, performed better than either NT-proBNP or miRNAs alone in sub-classifying PH.

**CONCLUSIONS:**

We suggest a threshold for NT-proBNP to identify patients with a high probability of PH, and the subsequent use of circulating miRNA signatures to help differentiate PH subgroups.

Pulmonary hypertension (PH), defined by a resting mean pulmonary artery pressure >20 mm Hg, is associated with reduced life expectancy.^1,2^ Patients present a diagnostic challenge,^3^ as their symptoms, such as shortness of breath, are not specific to an elevated pulmonary artery pressure, causing delays in diagnosis.^4^ Noninvasive investigations, such as transthoracic echocardiography and plasma brain natriuretic peptide (BNP) measurements, are used to identify patients for the definitive diagnostic test, right heart catheterization.^5,6^

Once PH is diagnosed, patients are assigned to 1 of 5 groups according to their clinical features and investigations, including but not exclusive to imaging, blood investigations and hemodynamic measurements^1^: namely, World Symposium on Pulmonary Hypertension (WSPH) Group 1: pulmonary arterial hypertension (PAH); WSPH Group 2: PH due to left heart disease (PH-LHD); WSPH Group 3: PH associated with lung disease (PH-Lung); WSPH Group 4: chronic thromboembolic PH (CTEPH); or WSPH Group 5: PH associated with unclear and multifactorial mechanisms (PH-miscellaneous). This clinical classification is used to guide management but takes limited account of complex patient phenotypes and comorbidities, which are increasingly common in the aging population.^7,8^

Circulating biomarkers (liquid biopsies ) have the potential to aid diagnosis and inform clinical management. To date, only BNP or its prohormone, NT-proBNP (N-terminal pro-B-type natriuretic peptide), has been adopted for risk stratification in European Society of Cardiology/European Respiratory Society guidelines.^1^ There are no established thresholds for the diagnosis of PH using BNP/NT-pro-BNP, and it is of limited diagnostic value when used in isolation as it does not discriminate between right and left ventricular strain or underlying causes of cardiac strain, having been originally described as a marker of cardiac stress in heart failure.^9,10^ Compounding this, it has limited sensitivity and is suboptimal as a screening tool for subclinical heart failure in the general population.^11^ To date, there have been no large-scale attempts to identify or validate new biomarkers that have utilized international prospectively collected data (collected under clinical trial conditions) that can aid diagnosis.

MicroRNAs (miRNAs) are nonprotein-coding sequences that have a critical role in regulating gene expression, with over 1000 miRNAs measurable in blood with high confidence.^12^ Previous studies have identified miRNAs as dysregulated in PH.^13–16^ Levels change with disease and may offer an alternative or be additive to BNP as a blood test to diagnose and risk stratify patients.^13,17^ Since miRNAs have a more diverse cellular origin and function than BNP, we hypothesized that the distribution of circulating miRNAs across the different presentations of PH would discriminate between PH subgroups, where NT-proBNP cannot.

## Methods

Detailed methods are provided in the Supplemental Material.

All data and code used to perform this analysis are available via the corresponding author and will be shared following appropriate governance and data sharing approval.

All samples and data were obtained following written informed consent to one of the following approved studies: the Imperial College Prospective Study of Patients with Pulmonary Vascular Disease cohort (UK Research Ethics Committee [REC] Ref 17/LO/0563), the Sheffield Teaching Hospitals observational study of PH, cardiovascular, and other respiratory diseases (STH-ObS, UK REC Ref 18/YH/0441), the Royal Papworth cohort (Cambridgeshire East REC Ref 08/H0304/56), or CIPHER study (A Prospective, Multicenter, Noninterventional Study for the Identification of Biomarker Signatures for the Early Detection of Pulmonary Hypertension, ClinicalTrials.gov: REGISTRATION: URL: https://www.clinicaltrials.gov; Unique identifier: NCT04193046).^18^

### Study Cohort Details

**Figure 1.**
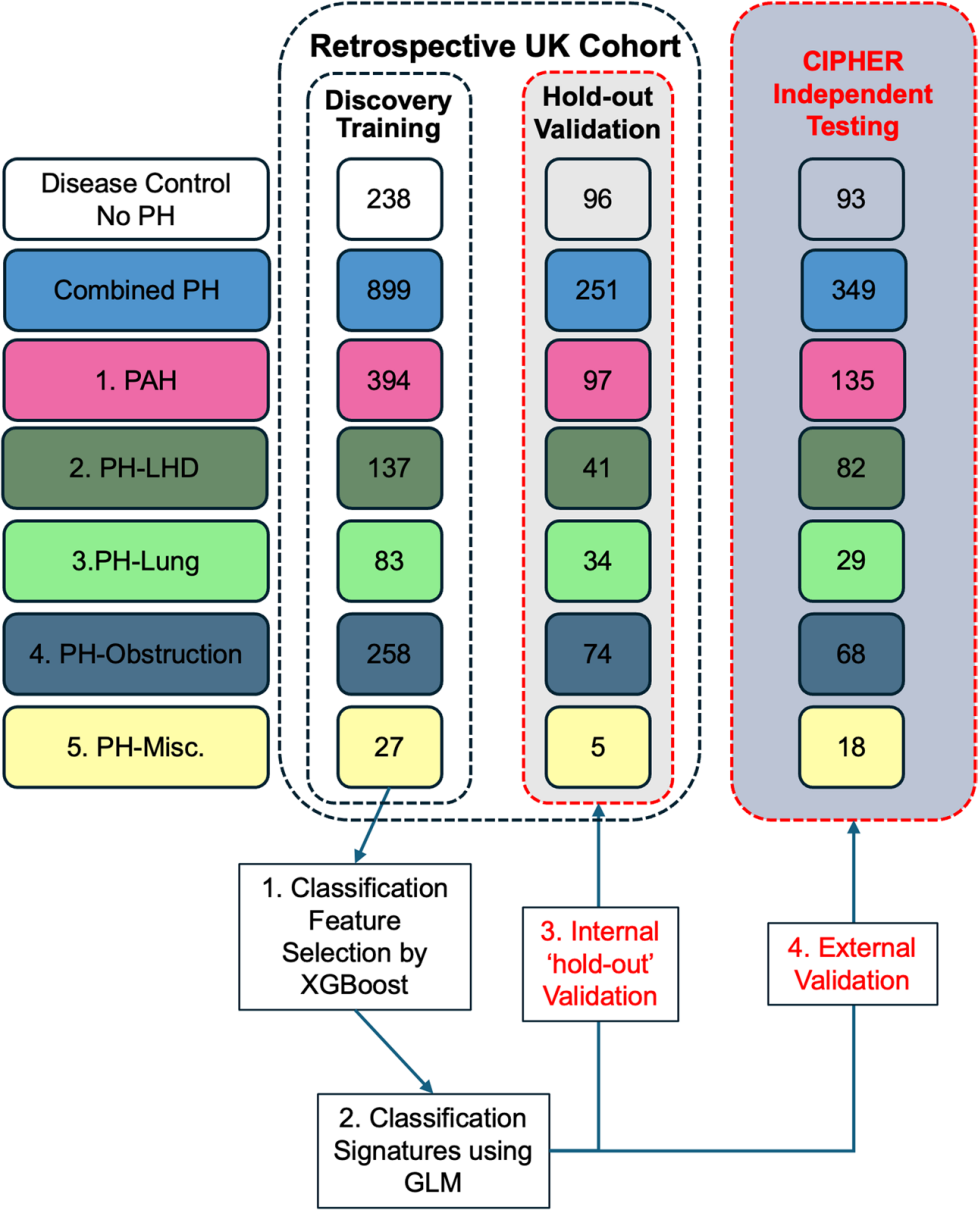
Study design. The combined number of patients with pulmonary hypertension (Combined PH), pulmonary arterial hypertension (PAH), PH due to left heart disease (PH-LHD), PH due to lung disease (PH-Lung), PH caused by pulmonary artery obstruction (PH Obstruction), and PH caused by miscellaneous factors (PH-Misc) subgroups and disease controls with no PH from the retrospective UK and prospective CIPHER (A Prospective, Multicenter, Noninterventional Study for the Identification of Biomarker Signatures for the Early Detection of Pulmonary Hypertension) Independent Test cohort are shown. The segregation of the Retrospective UK cohort into the Discovery/Training cohort and a Hold-out Validation cohort is shown. The boxes labeled 1 to 4 highlight the sequential steps taken for microRNA (miRNA) feature selection (1), miRNA signature model building using a generalized linear model (GLM; 2), internal testing in a held-out validation cohort (3), and independent external validation in the CIPHER study samples (4).

This study involved training and benchmarking classifiers for the various subgroups of PH using a retrospective cohort from the United Kingdom. After optimizing the classifiers, we tested them on the CIPHER study (A Prospective, Multicenter, Noninterventional Study for the Identification of Biomarker Signatures for the Early Detection of Pulmonary Hypertension), an independent validation cohort of international prospectively collected PH cases^18^ (Figure 1). The UK discovery cohort comprised 1150 patients with PH and 334 disease controls, split to training (n=1137 for model derivation) and hold-out validation (n=347 for model validation) groups, as summarized in Table 1; Table S1; Figure S1. Patients were recruited from 3 UK national PH referral centers, located at Hammersmith Hospital (Imperial College London), Royal Hallamshire Hospital (University of Sheffield), and Royal Papworth Hospital (Cambridge University), as summarized in Table S1. All cases were diagnosed between 2008 and 2019 using contemporaneous diagnostic guidelines.^19^ All samples were collected as per local standard operating procedures and stored at −80°C until assayed.

**Table 1.**
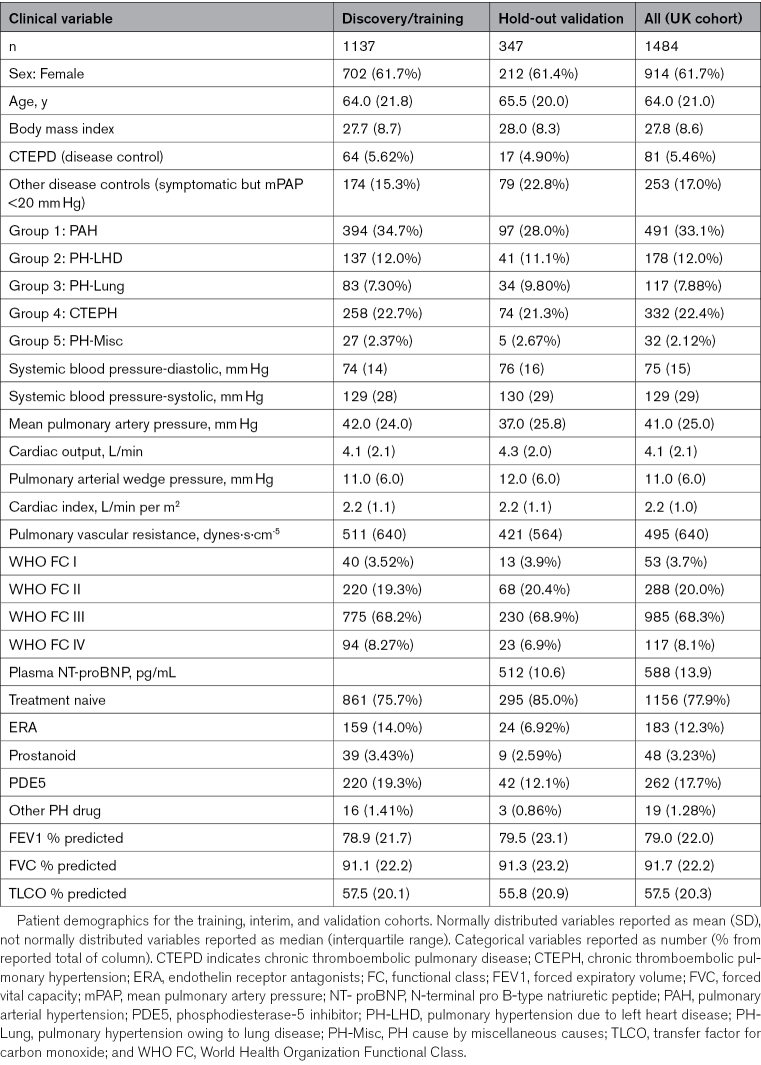
UK Retrospective Cohort Patient Demographics

### External Independent Validation

External validation was performed using data from CIPHER study (ClinicalTrials.gov: NCT04193046).^18^ CIPHER study enrolled participants from 44 sites across Belgium, France, Germany, the Netherlands, Poland, Spain, Ukraine, United Kingdom, and the United States of America between December 23, 2019, and December 20, 2021. The CIPHER protocol was reviewed and approved by the relevant institutional review board/independent ethics committee for each site and each patient gave written informed consent.^18^ None of the patients in the Retrospective UK cohort were also recruited into CIPHER study.

## Results

### Utility of NT-proBNP for Classifying PH and PH Subgroups

**Figure 2.**
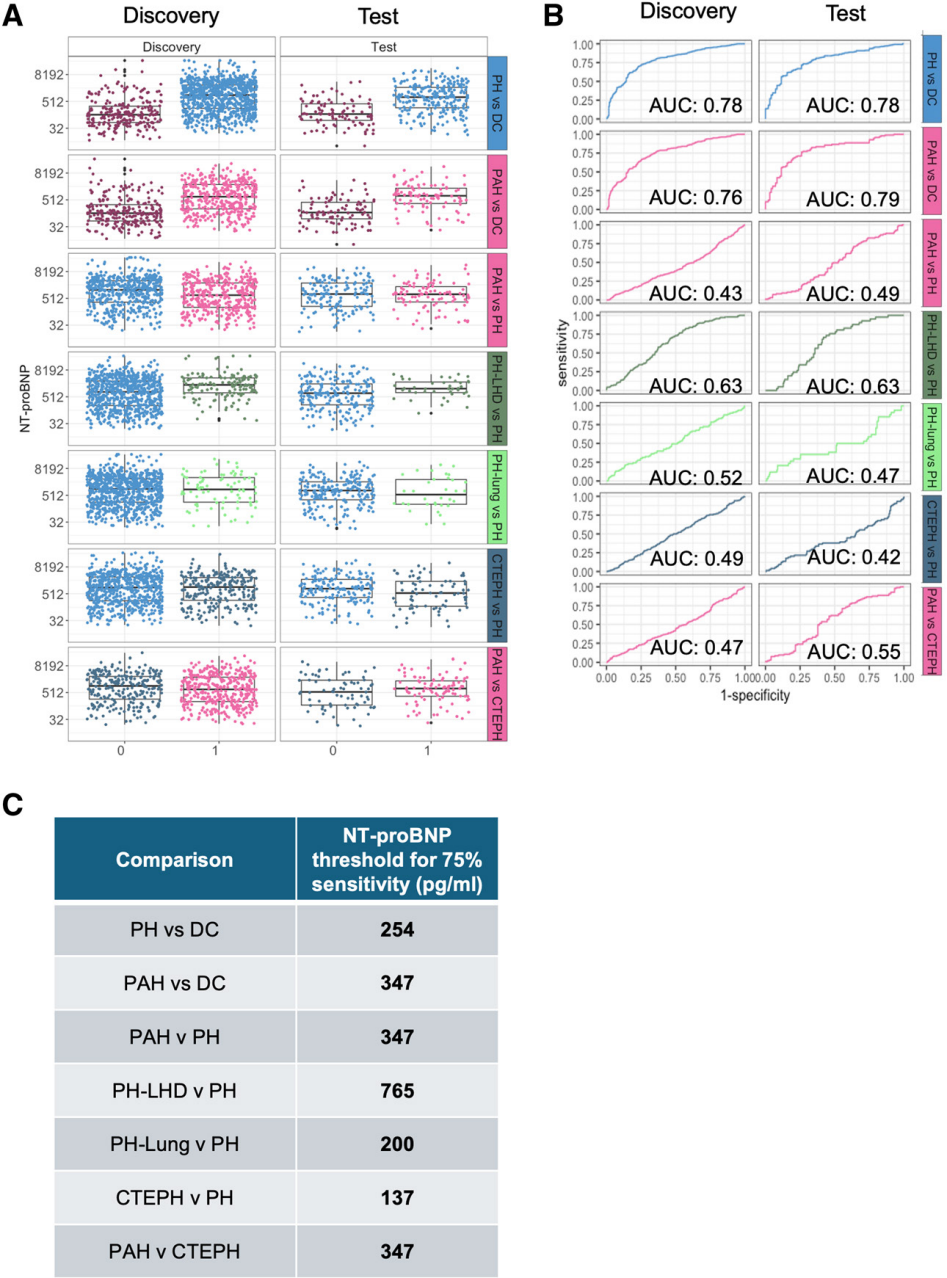
Utility of NT-proBNP (N-terminal pro-B-type natriuretic peptide) to classify pulmonary hypertension and subgroups. **A**, Box plots show the distribution of NT-proBNP expression in serum for combined pulmonary hypertension (PH), pulmonary arterial hypertension (PAH), PH due to left heart disease (PH-LHD), PH due to lung disease (PH-Lung), chronic thromboembolic PH (CTEPH) subgroups, and no PH disease controls (DCs) in the Discovery and Test populations from the retrospective UK cohort. **B**, Receiver operating characteristic (ROC) with area under the curve (AUC) for the use of NT-proBNP to classify PH and PAH from DC, and PAH, PH-LHD, PH-Lung, and CTEPH from a combined population of other PH groups trained on the Discovery and then tested on the internal UK hold-out Test population. **C**, Table shows the threshold of NT-proBNP derived from the UK Test population for a 75% sensitivity.

All clinical characteristics of the patient cohort are summarized in Table 1. The distribution of NT-proBNP levels in both the discovery and test cohorts for the DCs and all PH subgroups within the UK Retrospective Cohort is shown in Figure 2A (model generation described in the Supplemental Material and Tables S2 and S3). First, we tested the performance of NT-proBNP to classify PH from symptomatic disease controls (DCs), PAH from DC, and each PH subgroup (from all other forms of PH) in the UK retrospective cohort (Table S4). Cutoffs for NT-proBNP were determined based on values corresponding to 75% sensitivity in classifying the disease phenotype of interest from the United Kingdom hold-out test data. NT-proBNP performed well at distinguishing PH (area under the curve [AUC]=0.78) and PAH (AUC=0.79) from DC but was unable to differentiate PAH, PH-LHD, PH-Lung, or CTEPH (AUC=0.49, 0.63, 0.47, 0.42, respectively) from other forms of PH, or PAH from CTEPH (AUC=0.55; Figure 2B). Positive predictive values and negative predictive values are reported in Table S5. Based on these analyses, the optimal cutoffs for NT-proBNP for PH or PAH compared with DC were 254 and 347 pg/mL, respectively (Figure 2C). As expected, NT-proBNP performed poorly at sub-classifying PH subgroups from each other. NT-proBNP cutoffs for PAH versus PH and PAH versus CTEPH were identical to the cutoff for PAH versus DC (347 pg/mL), highlighting the limitations and lack of specificity for PH subgroups of NT-proBNP.

### Identifying miRNA Features to Build Signatures for PH and PH Subgroups

**Figure 3.**
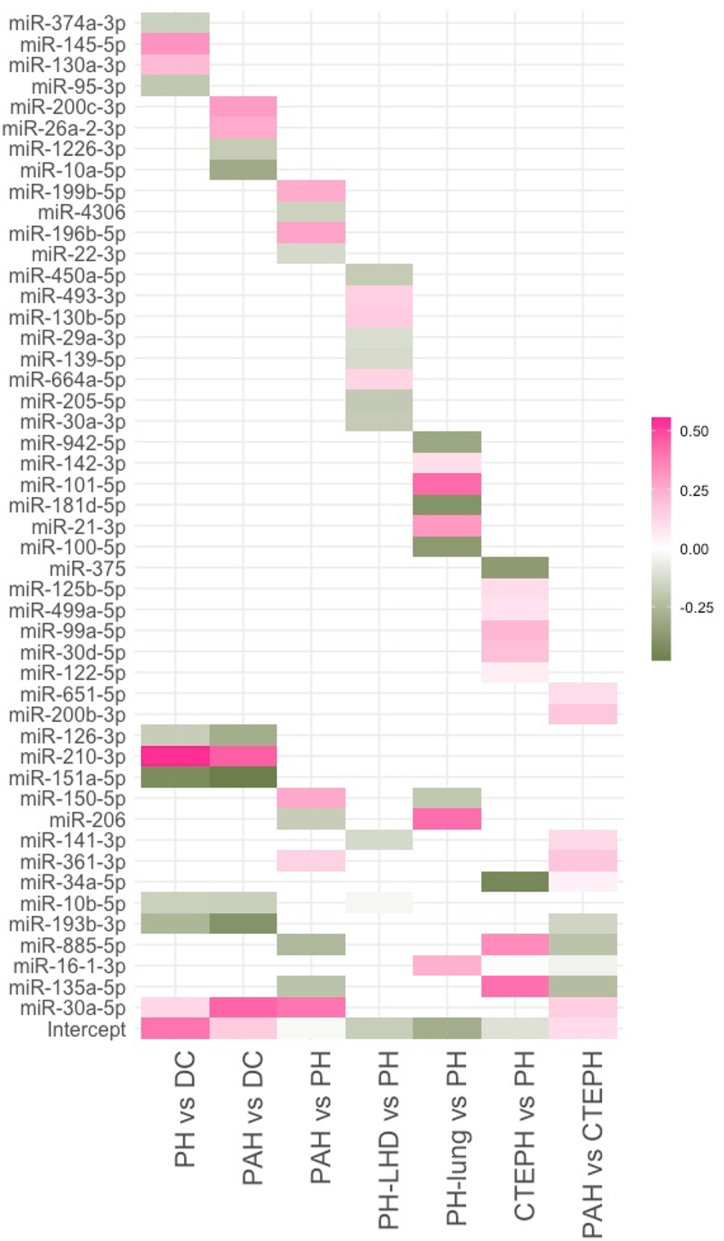
MicroRNA (miRNA) features selected for each signature and their coefficients. Heat map showing the miRNAs included in each pulmonary hypertension (PH), pulmonary arterial hypertension (PAH), PH due to left heart disease (PH-LHD), PH due to lung disease (PH-Lung), chronic thromboembolic PH (CTEPH) subgroups signatures, with their coefficients.

**Figure 4.**
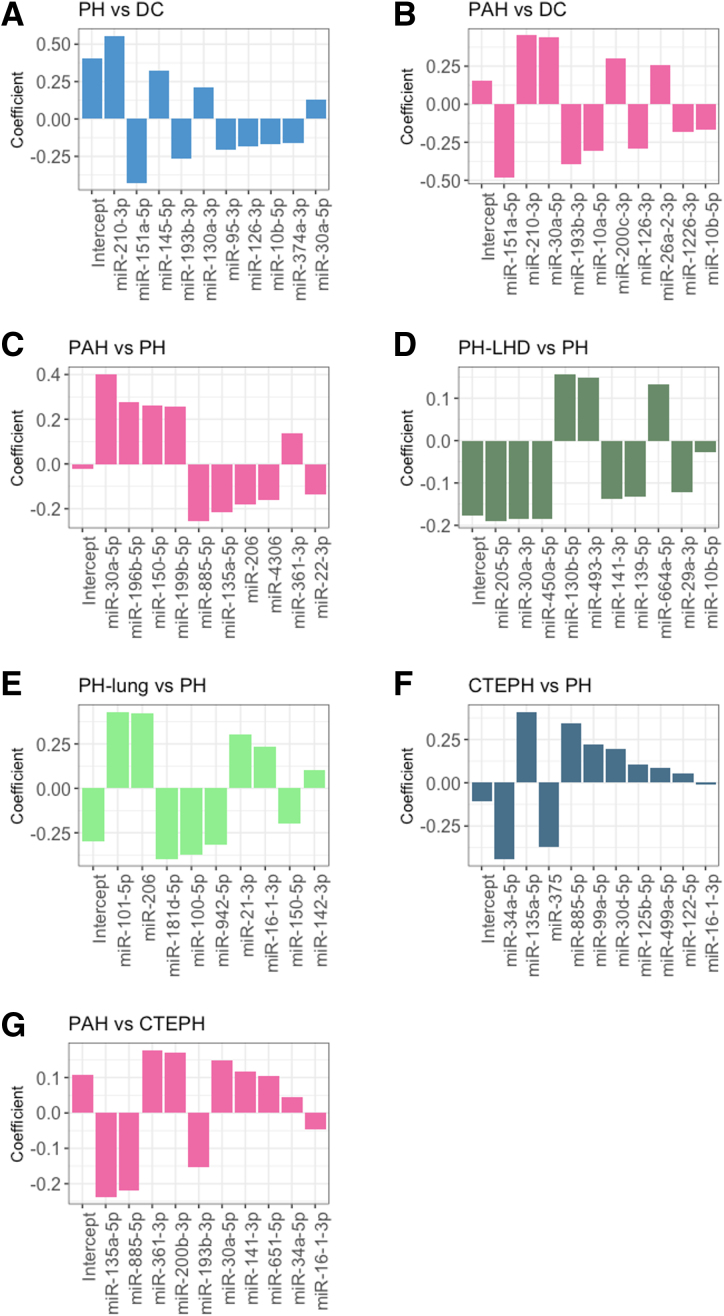
Pulmonary hypertension microRNA (miRNA) signature feature selection and coefficients from the UK Retrospective study. Histograms show the miRNAs selected for each signature and their coefficients within the generalized linear regression model used to classify (**A**) pulmonary hypertension (PH) vs no PH disease controls (DCs); (**B**) pulmonary arterial hypertension (PAH) vs DC; (**C**) PAH vs PH; (**D**) PH due to left heart disease (PH-LHD) vs PH; (**E**) PH due to lung disease (PH-Lung) vs PH; (**F**) chronic thromboembolic PH (CTEPH) vs PH; and (**G**) PAH vs CTEPH.

Given the limitations of NT-proBNP, we next tested whether we could identify distinct miRNA signatures for PH, and each PH subgroup. A unique signature containing a combination of 10 to 12 miRNAs was identified for each PH subgroup, with some common miRNAs between signatures (Figure 3). To improve interpretation of the classifiers and easy deployment to new patients and cohorts, we retrained a regression model using the selected features for each signature on the discovery cohort (Figure 4A through 4G). The signatures differentiating PH or PAH from DCs had the most similarity in terms of overlapping miRNAs but still contained 4 and 3 unique miRNAs, respectively (Figure 4A and 4B). Signatures to differentiate each of PAH, PH-LHD, PH-Lung, and CTEPH from the combined other forms (eg, PAH versus a combination of PH-LHD, PH-Lung, and CTEPH) contained largely unique miRNAs, and where there were overlaps, individual miRNAs had opposite coefficients (Figure 4C and 4F). Similarly, the miRNA signature to differentiate PAH from CTEPH contained miRNAs from the PAH and CTEPH (vs other PH) signatures but also contained some unique miRNAs (Figure 4G).

### Performance of miRNA Signatures in the UK Retrospective Cohort

**Figure 5.**
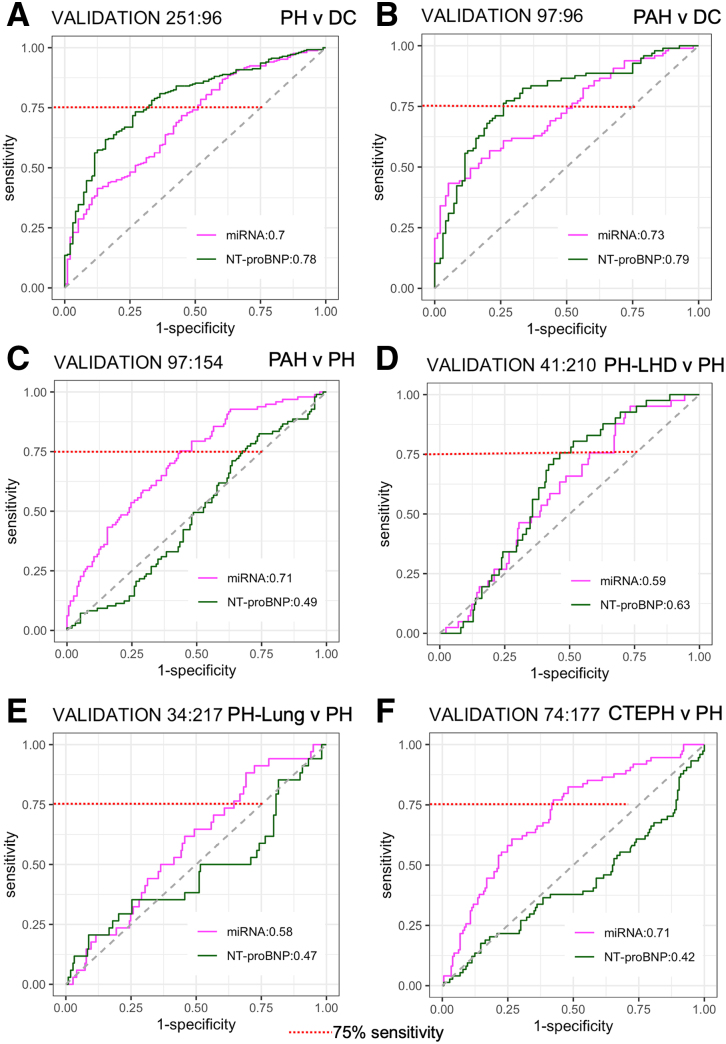
MicroRNA (miRNA) signature performance in the UK Hold-out Validation cohort. Receiver operating characteristic (ROC) with area under the curve (AUC) for the performance of the miRNA signatures (solid line) to classify (**A**) pulmonary hypertension (PH) vs no PH disease controls (DCs); (**B**) pulmonary arterial hypertension (PAH) vs DC; (**C**) PAH vs PH; (**D**) PH due to left heart disease (PH-LHD) vs PH; (E) PH due to lung disease (PH-Lung) vs PH; (**F**) chronic thromboembolic PH (CTEPH) vs PH compared with NT-proBNP (N-terminal pro-B-type natriuretic peptide; dashed line). *P* values from a DeLong test comparing the performance of the miRNA signature to NT-proBNP are shown for each. The dotted red line represents the 75% sensitivity threshold.

We performed internal validation on a hold-out set from the UK cohort. NT-proBNP achieved a slightly higher AUC than the miRNA signatures for classifying PH (AUC of 0.78 versus 0.70, *P*=0.03, Figure 5A; Table S4). There was no significant difference in the performance of the miRNA signature compared with NT-proBNP for the classification of PAH from DCs (AUC of 0.73 versus 0.79, *P*=0.23, Figure 5B; Table S4). In distinguishing PAH from other forms of PH, the miRNA signature significantly outperformed NT-proBNP (AUC of 0.71 versus 0.49, *P*<0.0001, Figure 5C; Table S4). However, there was no significant difference in the performance of the miRNA panel compared with NT-proBNP in classifying PH-LHD (AUC of 0.63 versus 0.59, *P*=0.56, Figure 5D; Table S4), or PH-Lung (AUC of 0.58 versus 0.47, *P*=0.14, Figure 5E; Table S4). For the classification of CTEPH from other forms of PH, the miRNA panel performed substantially better than NT-proBNP (AUC of 0.71 versus 0.42, *P*<0.0001, Figure 5F; Table S4). Similarly, the miRNA panel outperformed NT-proBNP in distinguishing PAH from CTEPH with a high AUC (AUC 0.76 versus 0.45, *P*<0.001, Table S4). The distribution of model scores in the discovery and UK validation cohorts is shown in Figures S1 and S2. The performance AUCs for each miRNA panel and NT-proBNP in the UK Discovery and Validation cohorts are summarized in Table S4.

### PH miRNA Signature Validation in the Independent CIPHER Cohort

To further validate these miRNA signatures in an external cohort, we obtained miRNA expression data from the prospective CIPHER study, where serum miRNA had been assayed using the same platform. All clinical characteristics of the patient cohort are summarized in Table 2. The expression of the miRNA panel constituents for each signature is shown in Figure S3. To test the performance of the signatures in classifying patients in CIPHER study, we used the cutoffs for each signature’s model score that achieved 75% sensitivity on the UK hold-out validation cohort (Figure 5). These cutoffs were then used in the external CIPHER cohort without modification. The addition of NT-proBNP alone to the model did not significantly improve the performance of the miRNA models (Figure S4). For distinguishing PH or PAH from DC, there was no significant difference in the accuracy of the miRNA panel compared with NT-proBNP (Table 3). However, for distinguishing PAH from other forms of PH, the miRNA signature significantly outperformed NT-proBNP (balanced accuracy of 0.58 versus 0.47, sensitivity of 0.74 versus 0.57, specificity of 0.43 versus 0.38, McNemar *P*=0.017, Table 3). NT-proBNP outperformed miRNA in classifying PH-LHD (balanced accuracy of 0.60 versus 0.52, sensitivity of 0.55 versus 0.70, specificity of 0.64 versus 0.34, *P*<0.0001, Table 3). There was no significant difference between the accuracy of the miRNA panel compared with NT-proBNP for the classification of PH-Lung (*P*=0.929, Table 3). However, for CTEPH, the miRNA panel significantly outperformed NT-proBNP, both in distinguishing CTEPH from other PH (balanced accuracy of 0.58 versus 0.47, sensitivity of 0.46 versus 0.75, specificity of 0.708 versus 0.19, *P*<0.0001), and from PAH (balanced accuracy of 0.68 versus 0.51, sensitivity of 0.69 versus 0.57, specificity of 0.68 versus 0.46, *P*=0.003, Table 3).

**Table 2.**
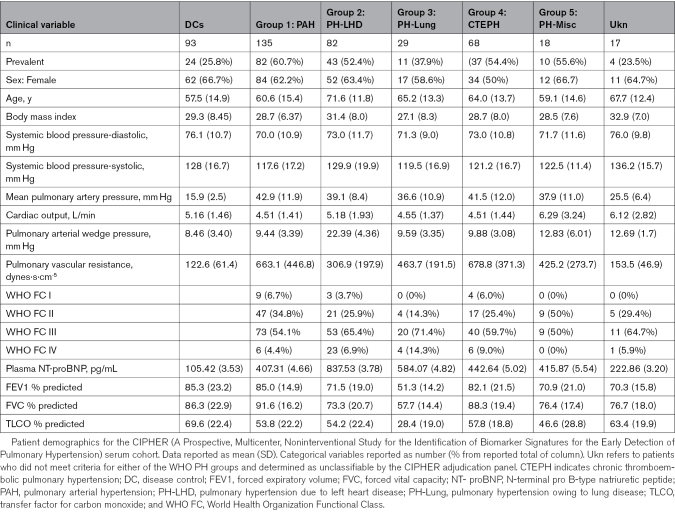
CIPHER Serum Cohort Patient Demographics

**Table 3.**
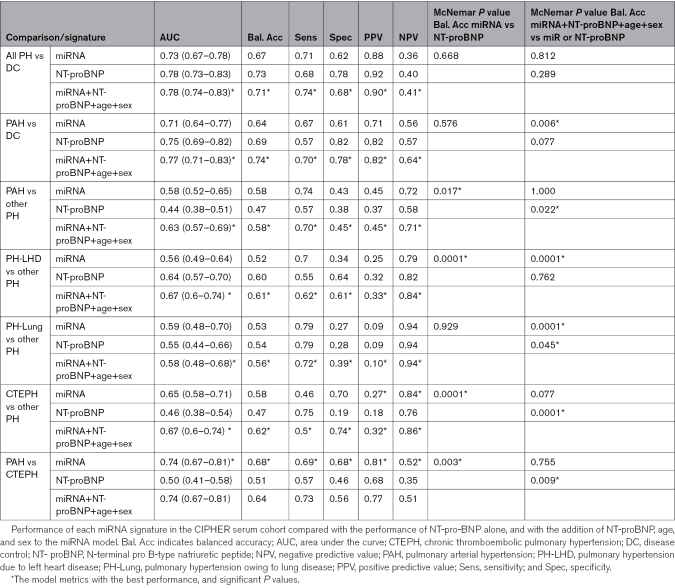
External Validation of UK miRNA Signature Performance in CIPHER Serum

**Figure 6.**
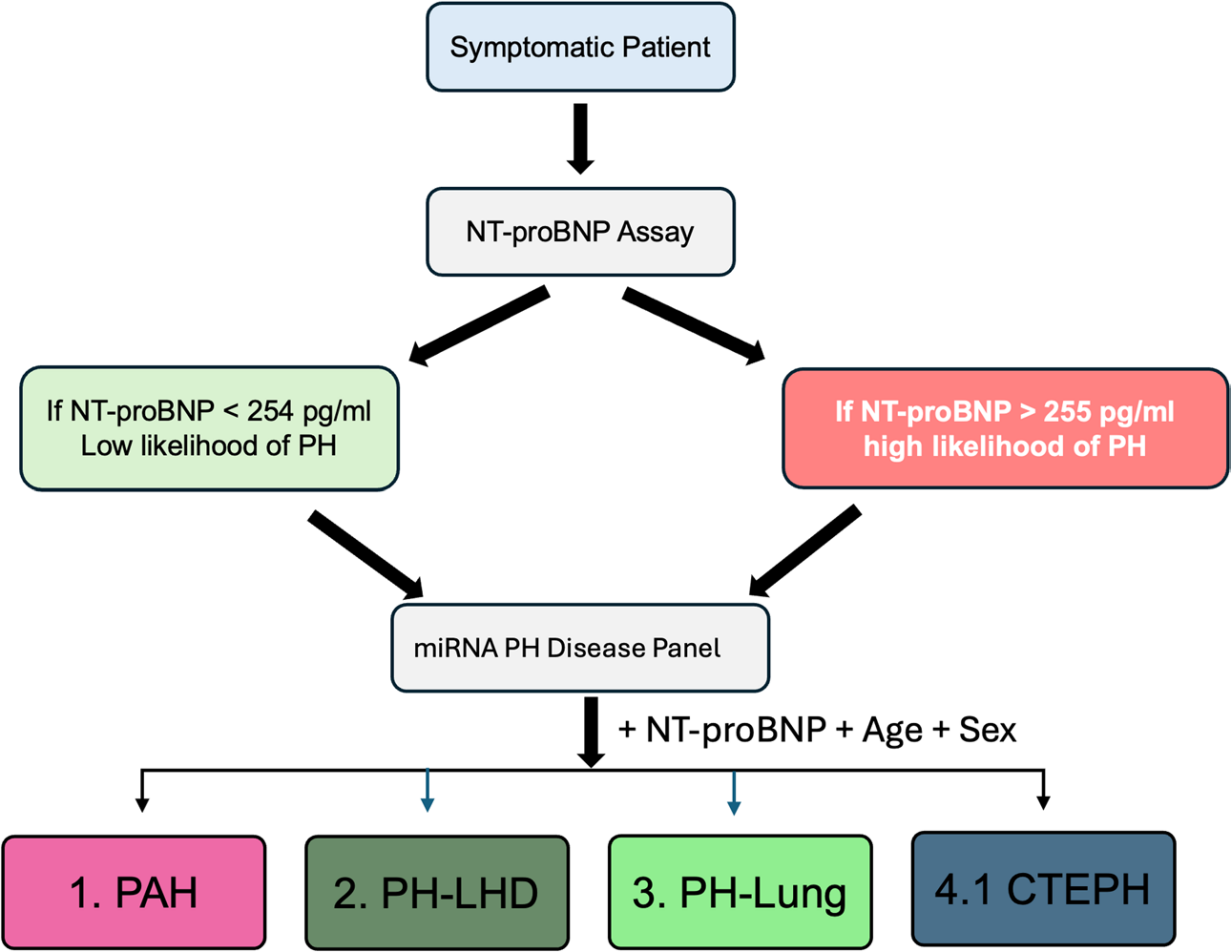
Flowchart and performance of UK microRNA (miRNA) diagnostic signatures in the CIPHER study. Flowchart summarizing the results obtained and highlighting the utility of miRNA signatures with NT-proBNP (N-terminal pro-B-type natriuretic peptide) to help guide the diagnosis of pulmonary hypertension (PH) and PH subgroups with blood-derived biomarkers from symptomatic patients to pulmonary arterial hypertension (PAH), PH due to left heart disease (PH-LHD), PH due to lung disease (PH-Lung), and chronic thromboembolic PH (CTEPH).

Since the addition of NT-proBNP to some miRNA panels improved performance, we next tested whether the combination of miRNA, NT-proBNP, along with age and sex, improved performance over the miRNA models alone. The performance of all additions to miRNA panel performance in the UK Validation cohort is shown in Table S5. Within the CIPHER Test cohort, for comparisons of PH to DC there was no significant improvement (or detriment) of the model over either the miRNA panel or NT-proBNP. In the PAH model, however, there was a significant improvement in performance compared with miRNA alone (balanced accuracy of 0.74 versus 0.64, sensitivity of 0.70 versus 0.67, specificity of 0.78 versus 0.61, *P*=0.006, Table 3; Figure S4). Similarly, there was a significant improvement in model performance with the inclusion of NT-proBNP, age, and sex for the WSPH subgroup signatures to identify PAH (balanced accuracy=0.58, sensitivity=0.70, specificity=0.45), PH-LHD (balanced accuracy=0.61, sensitivity=0.62, specificity=0.61), PH-Lung (balanced accuracy=0.56, sensitivity=0.72, specificity=0.39), CTEPH (balanced accuracy=0.62, sensitivity=0.50, specificity=0.74) from all other forms of PH there was a significant improvement compared with the miRNA panel alone (Table 3; Figure S4). The addition of NT-proBNP, age and sex had no significant improvement on the miRNA model to distinguish PAH from CTEPH. Finally, we tested whether PAH treatment affected the performance of the miRNA signatures by testing incident patients. There was no significant difference in any miRNA signature performance between incident and all cases of PH (Figure S5). The summary of how the miRNA panel can be used in conjunction with NT-proBNP, age, and sex to support PH diagnosis and classification is provided in Figure 6.

## Discussion

Here, we report the potential utility of circulating miRNAs to detect 2 subgroups of PH, namely PAH and CTEPH, for which there are licensed treatments. We utilized 2 large cohorts: a retrospective UK cohort from 3 expert PH centers consisting of 1484 patients (1150 with PH and 334 DCs) that were used to train, tune, and test miRNA signatures, and an independent testing comprising patients from the CIPHER^18^ (ClinicalTrials.gov: NCT04193046) clinical study.

We first used the cohorts to better understand the utility of NT-proBNP and suggest a specific diagnostic threshold for each comparison based on 75% sensitivity. To our knowledge, this has not previously been done, although NT-proBNP is used in various risk models^1,20,21^ and the DETECT algorithm.^22^ Our study has identified an NT-proBNP threshold level of 264 pg/mL for PH, which needs to be further validated in a prospective study. These data simultaneously highlighted known limitations of NT-proBNP as a non-PH (or PH subtype)-specific marker of right ventricle failure. This provided the platform to evaluate the utility of circulating miRNA levels.

A signature comprising 9 to 10 miRNAs matched but did not beat the performance of NT-proBNP in distinguishing PH or PAH from DC. However, separate miRNA signatures to identify WSPH subgroups of PH did outperform NT-proBNP, specifically in classifying patients with WSPH Group 1: PAH and WSPH Group 4.1: CTEPH from a mixed PH population of patients with all causes of PH. A further signature was also identified to distinguish PAH from CTEPH, which could be a useful addition to clinical imaging to determine disease etiology. The combination of miRNA signatures with NT-proBNP, age, and sex provided the best overall performance in detecting PH and PAH from DCs and distinguishing specific subgroups of PH from other patients with PH. However, the performance in distinguishing PH subgroups varied considerable in all metrics of sensitivity, specificity, and accuracy (0.56–0.71). While these may not yet be ready to support clinical decisions, they do represent a new category of PH biomarkers for PH subtypes that can be utilized as an important benchmark for future studies.

A change in circulating miRNA levels in patients with PH is well-recognized.^15–17^ The diseased pulmonary vascular bed offers a large surface area for the secretion and leakage of products into plasma. Previous studies have compared circulating miRNA levels in small cohorts of patients with idiopathic PAH and CTEPH with healthy controls, which limits the utility of the findings in clinical practice, where the goal is to identify subtypes of PH in a symptomatic population.^13,14^ By recruiting patients presenting to specialist clinics for diagnosis, we have been able to compare levels across the PH spectrum with DCs; specifically, patients with suspected PH where the condition has been excluded by right heart catheterization. This has resulted in miRNA signatures that hold potential for greater clinical value in the real-world diagnosis of PH.

Circulating miRNAs clearly carry more biological information than just reporting cardiac stress, the primary value of NT-proBNP measurements, and have the potential to better inform the molecular drivers of disease.^23^ Distinguishing PH-LHD and PH-lung from other forms of PH was particularly challenging, with signatures that did not fully validate either in the UK cohort or the CIPHER cohort; however, these were improved with the addition of NT-proBNP, age, and sex to the models. This difficulty may reflect the clinical heterogeneity (mild-severe PH) within these groups, and similarities in some of the reported underlying pathology. An overlap in the vascular histology of PAH and CTEPH has long been recognized.^24^ More recently, quantitative histomorphometry has shown global pulmonary vascular remodeling in the lungs of patients with PH-LHD^25^ and PH-Lung.^24^ Some commentators go as far as to suggest a pathology continuum between PAH and PH-LHD by describing atypical PAH.^26^ The structural remodeling observed in histology specimens is not confined to arterioles, and there is accumulating evidence supporting the involvement of the postcapillary pulmonary venous vasculature in all PH groups with varying degrees of intensity.^27^ We suggest that the challenges in developing specific WSPH Group 1 and 2 signatures might signal that these patients share some common pathology. Emerging evidence indicates that PH is a common cause of morbidity and mortality,^2,28,29^ and effective treatment relies on a better understanding of the molecular drivers behind different patient presentations. The challenges in identifying distinct signatures for PH-LHD and PH-Lung could suggest similarities in molecular mechanisms as well as pathology. This was emphasized by some overlapping miRNAs between signatures. This implies that treatment constrained by a clinical classification based largely on hemodynamic parameters may be limited and perhaps highlights the need for a more molecular-based classification. Future analysis of these miRNAs, and their targets may provide more insight into shared and distinct disease mechanisms.

This study represents one of the largest single-omic analyses of patients with suspected PH. We present an extensive biomarker study that suggests a PH-specific threshold for NT-proBNP, and highlights miRNA signatures that could help identify patients with subgroups of PH. Our rigorous procedure of validation and external testing of the signatures for PAH and CTEPH highlights the generalizability of the signatures, particularly in the real-world nature of sample collection within the CIPHER study (44 different PH centers). This is a significant strength of this study; however, despite its size, there are still insufficient numbers to examine a more granular subgroup analysis. The CIPHER study was initially conceived to develop a miRNA signature for PH, but it was not sufficiently powered to allow for both training and validation of miRNA signatures for PH subgroups as a standalone study. Yet, its heterogeneity has proved useful for external testing. With participants from 44 sites across Belgium, France, Germany, the Netherlands, Poland, Spain, Ukraine, United Kingdom, and the United States of America, the CIPHER study provided a diverse cohort on which to test prior-developed signatures, with all clinical diagnoses agreed upon by an adjudication panel to oversee consensus diagnosis for difficult cases^18^ to reduce the risk of contamination across WSPH subgroups. These diagnostic biomarker panels provide a new benchmark for future studies to improve upon and could be used to support earlier diagnosis by providing a test to elevate diagnostic risk and expedite referral, or with adjunct clinical information (already part of the ERS/ESC diagnostic algorithm, eg, electrocardiogram, history, x-ray^1^) in centers where right heart catheterization may not be available, although they are not suitable for use in isolation.

## ARTICLE INFORMATION

### Acknowledgments

The authors thank all the patients and their families who contributed to this research and the UK Pulmonary Hypertension Association for their support. The authors also acknowledge the contributions from all the health care professionals involved in generating the 3 cohorts: the Imperial College Prospective Study of Patients with Pulmonary Vascular Disease cohort (UK REC Ref 17/LO/0563), the Sheffield Teaching Hospitals observational study of pulmonary hypertension, cardiovascular, and other respiratory diseases (STH-ObS, UK REC Ref 18/YH/0441), and the Royal Papworth cohort (Cambridgeshire East Research Ethics Committee reference 08/H0304/56). We also acknowledge the valuable contributions from the patients enrolled and the practitioners supporting the CIPHER study (A Prospective, Multicenter, Noninterventional Study for the Identification of Biomarker Signatures for the Early Detection of Pulmonary Hypertension), which was supported by Actelion Pharmaceuticals, Ltd, a Johnson & Johnson Company, and all members of the CIPHER steering committee for their contributions.

### Sources of Funding

The authors gratefully acknowledge financial support from the UK Department of Health via the National Institute for Health Research comprehensive Biomedical Research Centre award to Imperial College Healthcare NHS Trust, Cambridge Biomedical Research Centre, the National Institute for Health Research Imperial Clinical Research Facility, and the National Institute for Health Research Biomedical Research Centre Sheffield Teaching Hospitals. The views expressed are those of the author(s) and not necessarily those of the National Institute for Health Research or the Department of Health and Social Care. Actelion Pharmaceuticals, a Johnson & Johnson Company. British Heart Foundation PG/11/116/29288, FS/15/59/31839 & FS/SBSRF/21/31025 (Dr Rhodes), FS/18/13/33281 (Dr Thompson), FS/18/52/33808 (Dr Lawrie), RE/18/4/34215 (Drs Wilkins, Errington, Lawrie).

### Disclosures

Drs Wilkins, Lawrie, Wang, Rhodes, Errington, Toshner, Fong, Jatkoe, He, and Lihan are named inventors in pending patent applications directed to aspects of this article. Drs Fong and Jatkoe were employees of Janssen Pharmaceutical Companies of Johnson & Johnson and own shares of stock/stock options in Johnson & Johnson. The other authors report no conflicts.

### Supplemental Material

Supplemental Methods

Tables S1–S5

Figures S1–S5

References 12,30

## Supplementary Material
