# Supplementary material for: Diagnostic MicroRNA Signatures to Support Classification of Pulmonary Hypertension
Source: Circ Genom Precis Med. 2025 Apr 18;18(3):e004862. doi: 10.1161/CIRCGEN.124.004862 (PMC12173163; doi:10.1161/CIRCGEN.124.004862)
Supplement: Supplementary file 1 [file hcg-18-e004862-s001.pdf]

SUPPLEMENTAL MATERIAL

Supplemental Methods

Study Design

This study involved training and benchmarking classifiers for the various subgroups of PH using a retrospective cohort from the UK. After optimising the classifiers, we tested them on CIPHER, an independent validation cohort of international prospectively collected PH cases[18] (Figure 1).

Retrospective UK Cohort (Discovery and Test)

The discovery cohort comprised 1150 patients with PH and 334 disease controls, split to training (n=1137 for model derivation), and hold-out validation (n=347 for model validation) groups as summarised in Table 1, Supplementary Table 1 and Figure 1. Patients were recruited from 3 UK national PH referral centres, located at Hammersmith Hospital (Imperial College London), Royal Hallamshire Hospital (University of Sheffield) and Royal Papworth Hospital (Cambridge University), as summarised in Supplementary Table 1. All cases were diagnosed between 2008 and 2019 using contemporaneous diagnostic guidelines[19]. All samples were obtained following written informed consent to one of three cohorts: the Imperial College Prospective Study of Patients with Pulmonary Vascular Disease cohort (PPVD, UK Research Ethics Committee [REC] Ref 17/LO/0563), the Sheffield Teaching Hospitals observational study of pulmonary hypertension, cardiovascular and other respiratory diseases (STH-ObS, UK REC Ref 18/YH/0441) or the Royal Papworth cohort (Cambridgeshire East REC Ref 08/H0304/56). All samples were collected as per local standard operating procedures and stored at -80°C until assayed.

To allow comparisons across each subgroup, samples were divided into a discovery and a hold-out test set in a 65:35 ratio. Samples were indexed by time from diagnosis/time on therapy and treatment-naïve with samples from each PH subgroup distributed evenly accounting for age and sex.

CIPHER Cohort (Independent Validation)

CIPHER (A Prospective, Multicenter, non-Interventional Study for the Identification of Biomarker Signatures for the early detection of Pulmonary Hypertension (PH), ClinicalTrials.gov - NCT04193046) was a prospective, non-therapeutic, multicenter study that primarily aimed to identify circulating microRNA (miRNA) biomarkers associated with PH and develop miRNA-based signatures to detect patients at high risk of PH[18]. CIPHER enrolled participants from 44 sites across Belgium, France, Germany, the Netherlands, Poland, Spain, Ukraine, United Kingdom and the United States of America between 23 December 2019 and 20 December 2021. None of the patients in the Retrospective UK cohort were also recruited into CIPHER. The study recruited new and follow-up adults who were referred to specialist PH clinics. The primary objectives of CIPHER involved developing a miRNA-based signature for detecting PH in patients with unexplained shortness of breath and estimating the performance of the miRNA biomarker signature and TTE in detecting PH, as measured against RHC, the gold-standard diagnostic test. This study utilized samples from 442 patients with 349 PH and 93 disease controls from whom serum was collected (Table 2). Patients within the CIPHER study underwent adjudication where there was discrepancy between the study criteria and supplied phenotype data as described in Lawrie et al 2024[18]. Briefly, cut-offs for FEV1 (greater than or equal to 60%) and FVC (greater than or equal to 70%) were applied to WSPH Group 1 PAH. Patients with FEV1 or FVC levels below this with a PAWP less than or equal to 15 were classified as WSPH Group 3.

Quantification of NT-proBNP and miRNAs

Total RNA was extracted from 200 µl of serum or plasma using the Maxwell® RSC miRNA Plasma and Serum Kit (Promega, Madison, USA) as per the manufacturer’s

recommendations with the following modifications: (a) a set of three proprietary spike-in controls (MiRXES, Singapore) was added, representing high, medium, and low levels of RNA, into the lysis buffer C prior to sample RNA isolation. The spike-in controls are 20-nucleotide RNAs with unique sequences (distinct from any of the 2588 annotated mature human miRNAs in miRBase version 21.0, RRID:SCR_003152) and are used to monitor RNA isolation efficiency and normalise for technical variations during RNA isolation; (b) bacteriophage MS2 RNA (Roche, Basel, Switzerland) was added at 0.4 ng per sample isolation to improve RNA isolation yield. For biomarker discovery, a highly controlled RT-qPCR workflow was used to quantify the expression of miRNA in each sample. Isolated RNA was reverse transcribed using miRNA-specific reverse transcription (RT) primers according to manufacturer's instructions (ID3EAL Customized Individual miRNA RT Primer, MiRXES) on QuantStudio™ 5 Real-Time PCR System (Applied Biosystems, Foster City, CA, USA).

The RT primers were divided into 12 multi-plex primer pools (20-60-plex per pool) to minimize non-specific cross-overs and primer-primer interactions. For each RNA sample, 12 multiplex RT reactions were performed, each with 5 μl of isolated RNA. Synthetic templates for standard curves of each miRNA (6-log serial dilution of 10 million to 100 copies) and a non-template control (nuclease-free water spiked with MS2) were reverse transcribed concurrently with the isolated sample RNA. Synthetic miRNA standard curves were used to absolutely quantify sample miRNA expression copy numbers. To measure miRNAs using quantitative PCR (qPCR), all cDNAs, including those from synthetic miRNA standards, were pre-amplified using a 15-cycle PCR reaction with Augmentation Primer Pools (MiRXES) on the QuantStudio™ 5 Real-Time PCR System according to manufacturer's instructions. Single-plex qPCR was then performed on the amplified cDNA samples using a miRNA-specific qPCR assay (MiRXES) and ID3EAL miRNA qPCR Master Mix according to manufacturer's instructions (MiRXES). The qPCR reactions with technical duplicates were carried out on the QuantStudio™ 5 Real-Time PCR System Raw threshold cycle (Ct) values were calculated using the QuantStudio™ Design and Analysis Software with automatic

baseline setting and a threshold of 0.4. RT-qPCR efficiency and potential cDNA amplification bias were assessed by analyzing the Ct values of the synthetic miRNA standards. The absolute expression of each miRNA (number of copies present) in the serum and plasma sample was calculated by intrapolation of sample Ct values with synthetic miRNA standard curves and correcting for variations in RT-qPCR efficiency.

Pre-processing of miRNA expression data

Data on 326 miRNAs detected in no less than 90% of samples were analysed further. Missing values were imputed separately in the discovery and subsequently the validation set. For a sample where a specific miRNA expression value was not detected, the expression value was set to the minimum value of that miRNA within 3 standard deviations of the mean across samples. For outliers where the expression value was more than 3 standard deviations above the mean, it was set to the maximum value within 3 standard deviations above the mean. miRNA data were further global normalized[30]. Samples from Cambridge showed higher total miRNA counts than the other centres. To further correct for this batch effect, total miRNA counts were modelled with a LASSO model composed of 11 miRNAs chosen from summed miRNA counts of detected miRNA by increasing the regularization parameter lambda arbitrarily to reduce selected miRNAs to a reasonable number with high performance (rho 0.9687) (Supplementary Table 2). Linear regression was then used to adjust the counts of each miRNA for any effect of the estimated total miRNA, retaining the mean miRNA levels.

miRNA feature selection

We utilised the Boruta package (v.7.0.0) using default settings with 300 iterations of the normalised permutation importance function using random forest to identify potentially relevant features within the classification framework. Once the 300 runs were completed, miRNAs not classified as important by the algorithm were rejected (p value threshold 0.01). This process was repeated 100 times, with miRNAs selected on at least 99 occasions

carried forwards into a random forest model, using the randomForest package (v4.6-14). The caret package was used to identify the optimal number of trees, from 1000, 1500, 2000 or 2500. The number of variables available for splitting at each tree node was optimised next, across the range 1 to 15. Once a model was created, if the original number of variables selected was greater than 10, the model was re-tuned by removing the variables with the lowest attributed importance to the model.

Recursive partition trees were computed using Rpart (v4.1-15 http://CRAN.R-project.org/package=rpart) and caret in R. Trees were generated from the root of the tree downwards, using a greedy feature selection algorithm and recursive binary splitting to return features in order. The tree construction was controlled by setting the minimum number of observations in a terminal node to 5 and the minimum number of observations in a node for a split to occur set to 4. The Gini index was minimised to create each split. A variable was deemed to be selected if it appeared in the final model.

Least absolute shrinkage and selection operator (LASSO) using the glmnet package in R (v4.1-2) was used to choose pertinent miRNAs by eliminating variables with a coefficient shrunk to 0. The regularisation parameter, λ, was chosen using 10-fold cross-validation with binomial deviance as the criterion. The value of λ with minimum binomial deviance was selected and used to fit the final model.

XGBoost (v1.4.1.1) parameters were tuned over previously described optimisation ranges for miRNAs (Errington et al. 2021). Once models had been trained, the variables were ranked in terms of their importance. The top 9 miRNAs were then selected, and the model re-trained over the same parameter range using only the 9 selected miRNAs. The optimal values for these parameters for each model can be seen in Supplementary Table 3. Multiple analytic approaches were only used in the initial features selection process.

Training and testing of classification models generated in UK Retrospective Cohort

The selected miRNA features were trained by ridge regression (α=0) using *glmnet* package in R on the discovery cohort. Ridge regression was chosen to reduce over-fitting by controlling the strength of regularization. The optimal regularization parameter λ was obtained by using 10-fold cross-validation and optimising on the area under the receiver operating characteristic curve (AUC). The value of λ with maximum AUC was selected and used to fit the final model. Coefficients for each miRNA from the fitted ridge regression model were obtained.

Testing the performance of UK miRNA signatures on the CIPHER study

For model score calculation, standardized (Z-scored) miRNA values were multiplied by the coefficients obtained from UK model, summed up to get logit (L), which was then transformed into probability by this formula 1/(1+exp(-L)). While the model was built using the full panel of miRNAs, for testing only miRNAs that were detected in CIPHER were entered into the model to calculate the model score. Any missing miRNA did not contribute to the score. For PH vs DC, all 10 miRNAs are detectable in the CIPHER dataset. For the PH-Lung vs PH analysis, 8 out of 9 miRNAs, and for all other comparisons, 9 out of 10 miRNAs were detected in CIPHER. Despite these missing miRNAs, the performance of the regression models (not re-trained for the missing miRNA) were tested on the CIPHER cohort. For each miRNA signature, we classified a sample as positive for a class if the model's score was greater than or equal to the threshold selected from tests on the UK cohort that achieved 75% sensitivity. The predicted class labels were then compared to the ground truth labels to construct a confusion matrix. This matrix was used to calculatebalanced accuracy, sensitivity, and specificity FPP, NPV of each model alone, in comparison to NT-proBNP alone, and in combination with NT-proBNP, age and sex. The DeLong test from *pROC* package (v1.18.4) was used to compare AUCs from the miRNA and NT-proBNP models. AUC, PPV, NPV and sensitivity were calculated to compare performance as defined[31].

Performance of NT-proBNP as a PH Classifier

We also investigated the performance of NT-proBNP as a standalone variable for classification. The glm function in R was used to build binary logistic regression models for each comparison.

Supplemental Tables

Table S1: UK Retrospective Cohort - Demographic data by clinical classification group

Clinical Variable	DC	PAH	PH-LHD	PH-Lung	CTEPH	Misc-PH
n	334	491	178	117	332	32
Sex: Female (%)	221 (66.2)	351 (71.5)	106 (59.6)	58 (49.6)	165 (49.7)	13 (40.6)
Age (years)	63.0 [22.7]	61.0 [24.0]	74.0 [12.0]	68.0 [12.0]	64.0 [12.0]	62.6 [18.2]
Body Mass Index	28.4 [7.40]	27.1 [8.09]	29.3 [10.6]	27.1 [9.68]	28.2 [8.19]	25.2 [6.12]
**Haemodynamics**						
Blood pressure - diastolic (mm Hg)	75.5 [15.0]	73.0 [15.0]	74.0 [17.0]	75.5 [15.2]	76.0 [17.0]	72.0 [10.8]
Blood pressure - systolic (mm Hg)	134 [27.2]	122 [25.2]	141 [29.0]	130 [28.0]	128 [27.0]	122 [22.0]
Pulmonary Vascular Resistance (dynes)	160 [86.4]	752 [679]	271 [233]	480 [454]	629 [496]	480 [448]
Mean pulmonary	20.0 [5.00]	48.0 [17.5]	36.0 [14.2]	39.5 [18.2]	44.5 [20.0]	45.0 [14.5]

artery pressure (mm Hg)						
Cardiac Output (L/min)	5.11 [2.04]	3.90 [2.06]	4.20 [1.99]	4.12 [2.14]	4.07 [1.08]	4.40 [2.29]
Cardiac Index (L/min/m2)	2.50 [1.07]	2.18 [1.16]	2.18 [1.08]	2.47 [1.08]	2.14 [0.77]	2.57 [1.11]
SVO2 (%)	71.5 (6.78)	65.0 (10.6)	64.4 (8.48)	64.7 (10.5)	62.7 (9.73)	62.7 (11.0)
**WHO Functional Class**						
I	34 (10.1%)	11 (2.2%)	2 (1.2%)	0 (0%)	6 (1.8%)	0 (0%)
II	109 (32.6%)	82 (16.7%)	21 (11.8%)	12 (10.3%)	59 (17.8%)	5 (15.6%)
III	182 (54.5%)	328 (66.8%)	140 (78.7%)	71 (60.7%)	244 (73.5%)	20 (62.5%)
IV	5 (2.7%)	51 (10.4%)	8 (4.5%)	29 (24.8%)	17 (5.1%)	7 (21.9%)
Plasma NT-proBNP (log2 pg/ml)	7.07 [2.50]	9.56 [3.42]	10.7 [2.12]	9.64 [3.81]	9.68 [3.71]	10.6 [4.25]
PH Treatment naive	331 (99.1%)	266 (54.2%)	173 (97.2%)	112 (95.7%)	249 (75.0%)	25 (78.1%)
**Pulmonary Function Test**						
FEV1 % Predicted	88.3 (21.7)	82.3 (20.9)	71.6 (22.4)	60.1 (23.9)	79.5 (16.1)	67.4 (17.6)
FVC % Predicted	97.6 (22.1)	92.2 (21.5)	83.0 (23.1)	79.4 (24.7)	95.2 (19.4)	80.5 (18.5)
TLCO % Predicted	67.7 (19.1)	51.7 (21.8)	58.2 (17.9)	34.5 (17.2)	61.3 (14.9)	50.4 (19.9)

Table S2: LASSO coefficients for calculating total miRNA counts

miRNA	Coefficient
Intercept	2372.120544
hsa-miR-106b-3p	26.643133
hsa-miR-652-3p	4.090500
hsa-miR-128-3p	0.739706
hsa-miR-148b-3p	8.934031
hsa-miR-328-3p	6.758305
hsa-miR-340-5p	33.798210
hsa-miR-1271-5p	4.647773
hsa-miR-30d-5p	22.900814
hsa-miR-222-3p	18.635122
hsa-miR-324-5p	10.632438
hsa-miR-425-5p	27.380878

Table S3: Final XGBoost model parameters for each comparison.

Comparison	No of trees	Max tree depth	Learning rate	Gamma	% feature used in each boost (column sampling)	Min child weight	Subsample rate (row sampling)
PH vs DC	9950	10	0.01	0	1	1	1
PAH vs DC	1500	2	0.01	0	1	2	1
PAH vs other PH	3950	1	0.025	0	1	1	1
PH-LHD vs other PH	9100	6	0.01	0.5	0.4	1	1
PH-lung vs other PH	5400	7	0.025	0	0.6	1	1
CTEPH vs other PH	200	3	0.1	0	1	5	1
PAH vs CTEPH	2550	1	0.01	0.05	0.6	2	0.75

Table S4: Performance of miRNA signatures in discovery and test datasets from the UK cohorts

**Comparison**	**UK Discovery miRNA AUC (95% CI)**	**UK Discovery NT-proBNP AUC (95% CI)**	**UK Test miRNA AUC (95%CI)**	**UK Test NT-proBNP AUC (95% CI)**	**UK Test DeLong test p value***
All PH vs DC	0.76 (0.73-0.79)	0.78 (0.75-0.82)	0.70 (0.64-0.76)	0.78 (0.73-0.84)	0.0067
PAH vs DC	0.81 (0.77-0.84)	0.76 (0.72-0.79)	0.73 (0.66 – 0.80)	0.79 (0.72 – 0.85)	0.1445
PAH vs other PH	0.71 (0.68-0.74)	0.43 (0.40-0.47)	0.71 (0.65-0.77)	0.49 (0.44-0.56)	0.0001
PH-LHD vs other PH	0.74 (0.70-0.79)	0.63 (0.59-0.68)	0.59 (0.51 – 0.68)	0.63 (0.55 - 0.70)	0.4954
PH-lung vs other PH	0.73 (0.68-0.78)	0.52 (0.45-0.59)	0.58 (0.49 - 0.68)	0.47 (0.35 - 0.59)	0.1079
CTEPH vs other PH	0.70 (0.66-0.73)	0.49 (0.45-0.54)	0.71 (0.63-0.78)	0.42 (0.34-0.50)	0.0001
PAH vs CTEPH	0.72 (0.68-0.76)	0.47 (0.42-0.52)	0.76 (069	0.55 (0.46-0.64)	0.0001

*Classification AUC on the discovery and UK validation data sets for the miRNAs and NT-proBNP glm models across five clinically defined classes of Pulmonary Hypertension and Disease Control). PH, pulmonary hypertension; PAH, pulmonary arterial hypertension; PH-LHD, pulmonary hypertension due to left heart disease; PH-Lung, pulmonary hypertension due to lung disease; CTEPH, chronic thromboembolic pulmonary hypertension; DC, disease controls. *P value for DeLong test of miRNA and NT-proBNP models on the validation set.*

Table S5: Positive predictive values and negative predictive values for the performance of glm models in the UK validation cohort.

**Comparison**	**Model**	**Bal. Acc**	**Sensitivity**	**Specificity**	**PPV**	**NPV**
PH vs DC	miRNA	0.62	0.76	0.49	0.79	0.78
PH vs DC	NT-proBNP	0.72	0.76	0.68	0.86	0.56
PH vs DC	miRNA+ NT-proBNP	0.72	0.76	0.68	0.86	0.91
PH vs DC	miRNA + age + sex	0.62	0.76	0.48	0.79	0.93
PH vs DC	miRNA + NT-proBNP + age + sex	0.70	0.76	0.65	0.85	0.80
PAH vs DC	miRNA	0.62	0.75	0.48	0.59	0.85
PAH vs DC	NT-proBNP	0.75	0.75	0.74	0.74	0.93
PAH vs DC	miRNA+ NT-proBNP	0.71	0.75	0.68	0.70	0.52
PAH vs DC	miRNA + age + sex	0.64	0.76	0.51	0.61	0.68
PAH vs DC	miRNA + NT-proBNP + age + sex	0.76	0.76	0.76	0.76	0.73
PAH vs PH	miRNA	0.65	0.75	0.54	0.51	0.66
PAH vs PH	NT-proBNP	0.54	0.75	0.32	0.41	0.85
PAH vs PH	miRNA+ NT-proBNP	0.62	0.75	0.49	0.48	0.68
PAH vs PH	miRNA + age + sex	0.68	0.76	0.61	0.55	0.43
PAH vs PH	miRNA + NT-proBNP + age + sex	0.68	0.76	0.60	0.54	0.80
PH-LHD vs PH	miRNA	0.57	0.76	0.38	0.19	0.44
PH-LHD vs PH	NT-proBNP	0.64	0.76	0.51	0.23	0.55
PH-LHD vs PH	miRNA+ NT-proBNP	0.61	0.76	0.47	0.22	0.78
PH-LHD vs PH	miRNA + age + sex	0.67	0.76	0.58	0.26	0.66
PH-LHD vs PH	miRNA + NT-proBNP + age + sex	0.69	0.76	0.62	0.28	0.89
PH-lung vs PH	miRNA	0.56	0.76	0.35	0.15	0.75
PH-lung vs PH	NT-proBNP	0.48	0.76	0.19	0.13	0.52
PH-lung vs PH	miRNA+ NT-proBNP	0.57	0.76	0.38	0.16	0.92
PH-lung vs PH	miRNA + age + sex	0.61	0.76	0.47	0.18	0.67
PH-lung vs PH	miRNA + NT-proBNP + age + sex	0.57	0.76	0.39	0.16	0.84
CTEPH vs PH	miRNA	0.67	0.76	0.58	0.43	0.93
CTEPH vs PH	NT-proBNP	0.43	0.76	0.11	0.26	0.80
CTEPH vs PH	miRNA+ NT-proBNP	0.67	0.76	0.58	0.43	0.86
CTEPH vs PH	miRNA + age + sex	0.67	0.75	0.59	0.44	0.91
CTEPH vs PH	miRNA + NT-proBNP + age + sex	0.69	0.75	0.62	0.45	0.68
PAH vs CTEPH	miRNA	0.69	0.75	0.64	0.73	0.91

PAH vs CTEPH	NT-proBNP	0.57	0.75	0.39	0.62	0.91
PAH vs CTEPH	miRNA+ NT-proBNP	0.67	0.75	0.59	0.71	0.65
PAH vs CTEPH	miRNA + age + sex	0.72	0.76	0.67	0.75	0.85
PAH vs CTEPH	miRNA + NT-proBNP + age + sex	0.72	0.76	0.68	0.76	0.51

Performance metrics for the glm model in the UK validation cohort. PPV; positive predictive value, NPV; negative predictive value.

Supplemental Figures

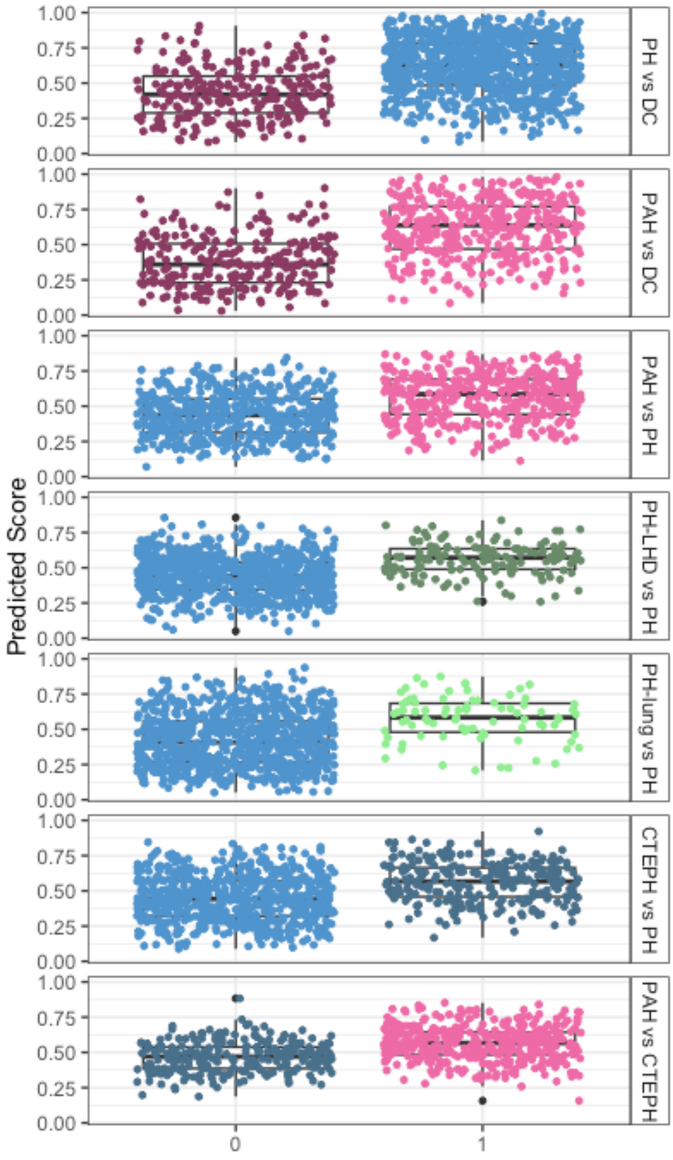

Figure S1: miRNA signature model score from the UK Discovery cohort. Box and Whisker plots showing the model score for each miRNA signature in the Discovery cohort.

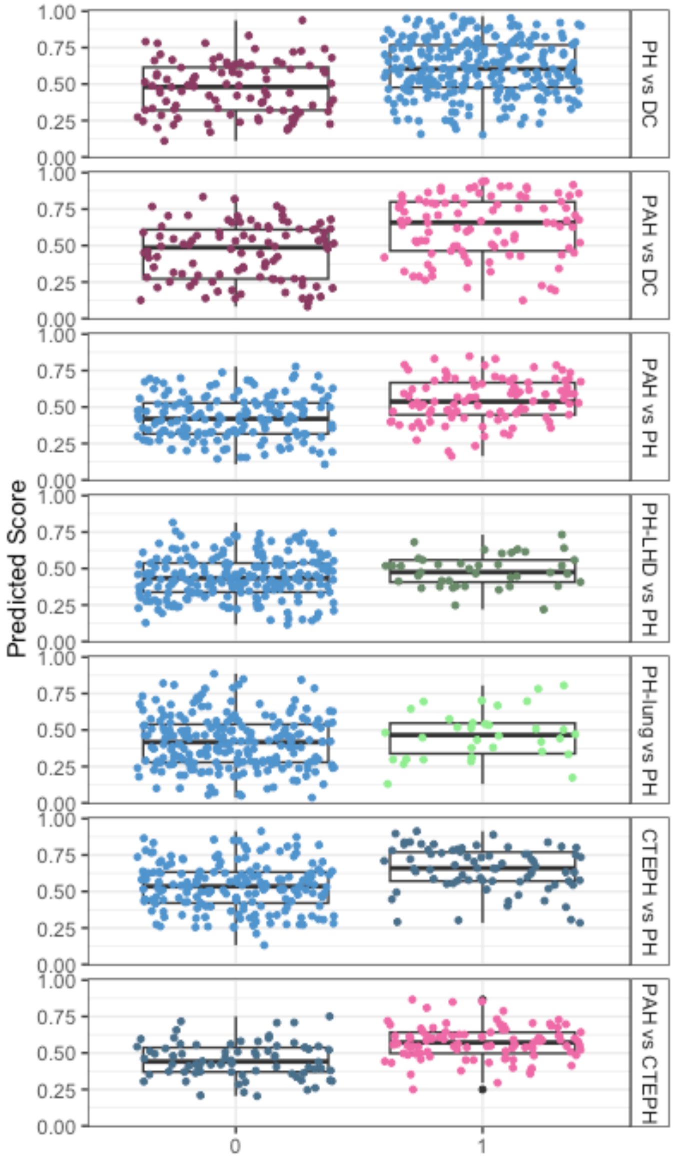

Figure S2: miRNA signature model score from the UK Validation cohort. Box and Whisker plots showing the model score for each miRNA signature in the Test cohort using cut-offs obtained from the Discovery cohort.

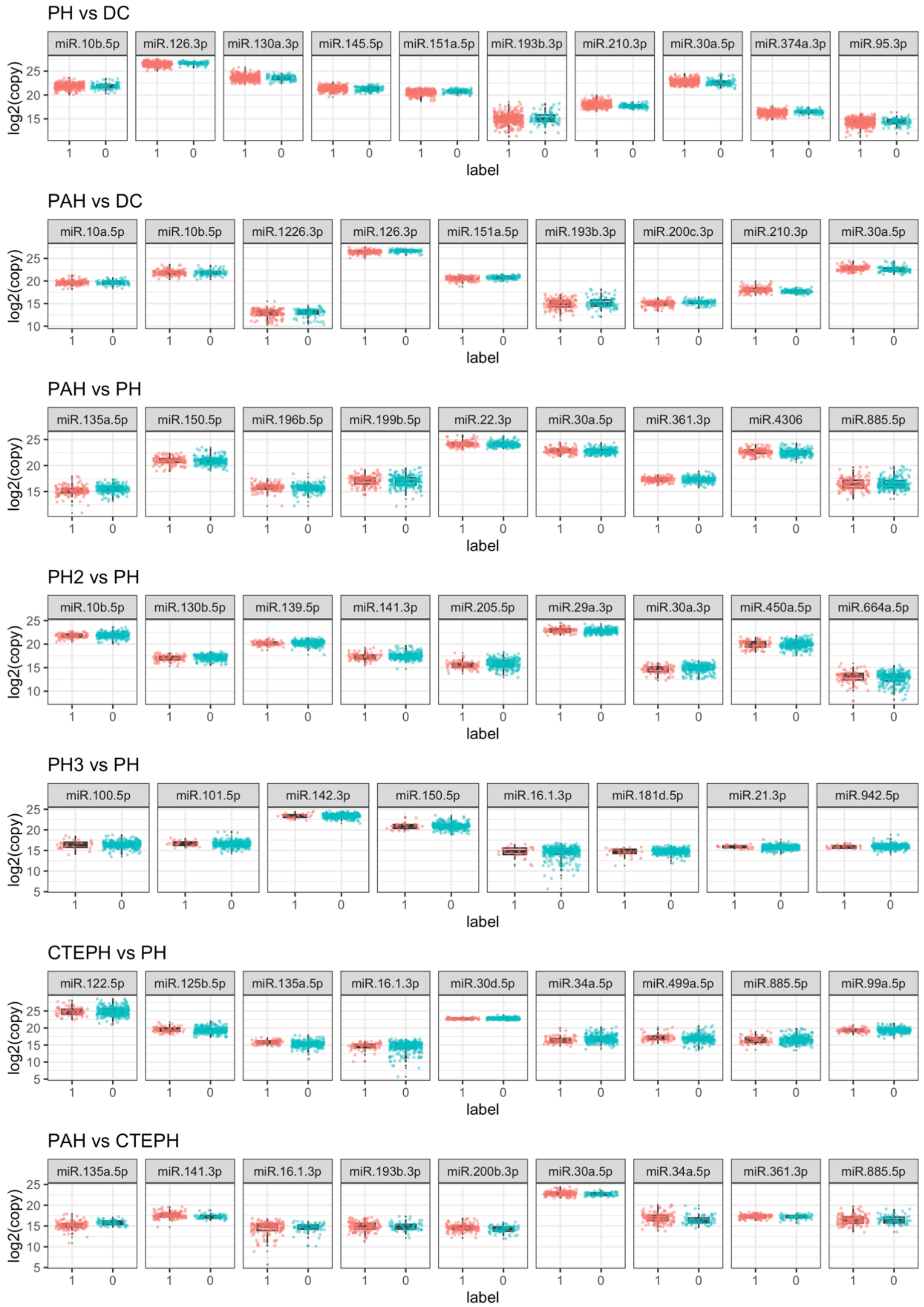

Figure S3: miRNA signature constituent expression in the CIPHER serum sub-study.

Box and Whisker plots showing the expression of each miRNA from the miRNA signatures in for each comparison.

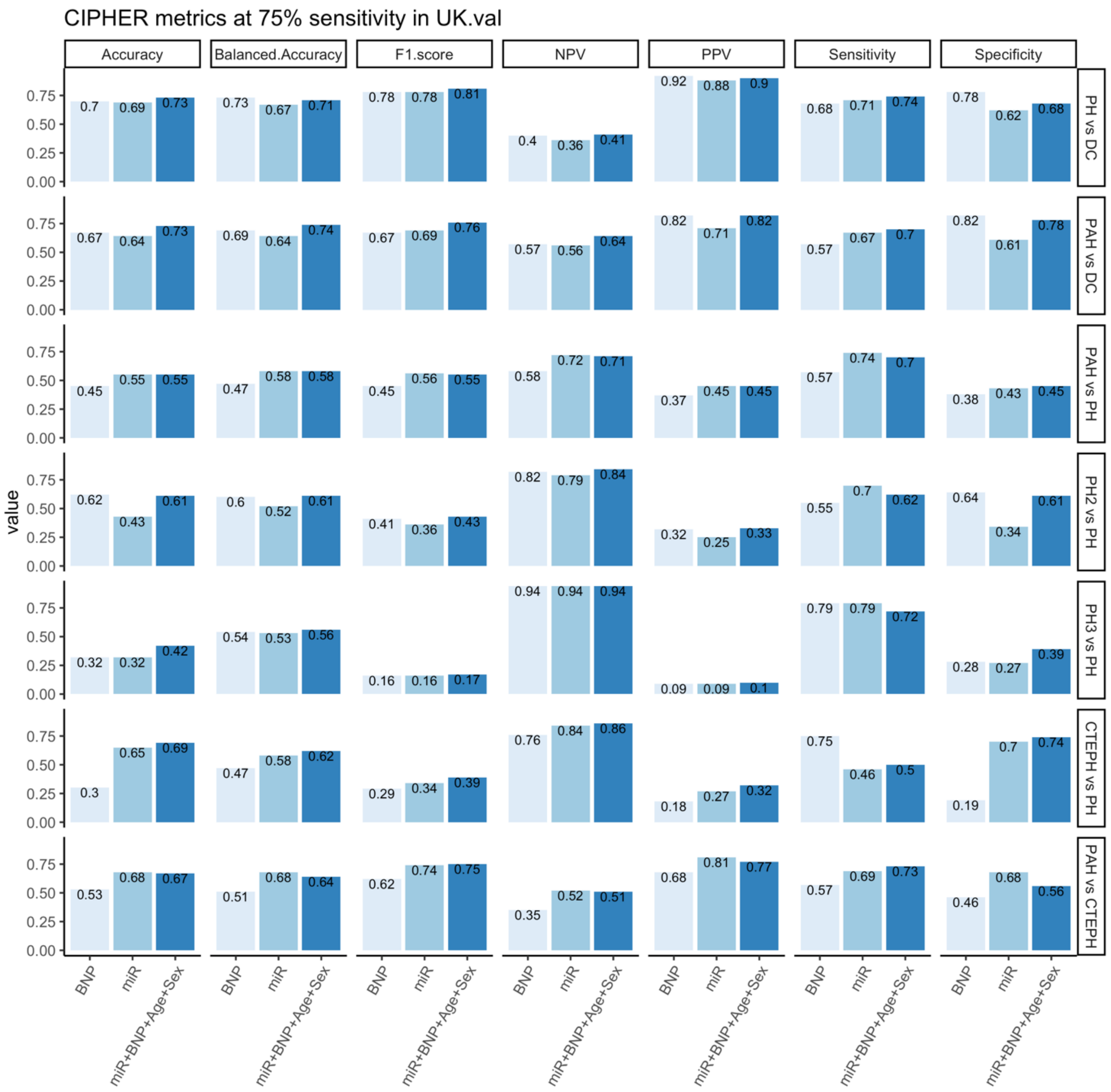

Figure S4: miRNA signature model performance in the CIPHER serum sub-study.

Histograms show the Balanced accuracy with sensitivity and specificity for each miRNA signatures alone, in comparison to NT-proBNP alone, and in combination with NT-proBNP.

A) Model performance in UK test data reveals no difference between treatment naive and all PH patients with each WSPH group.

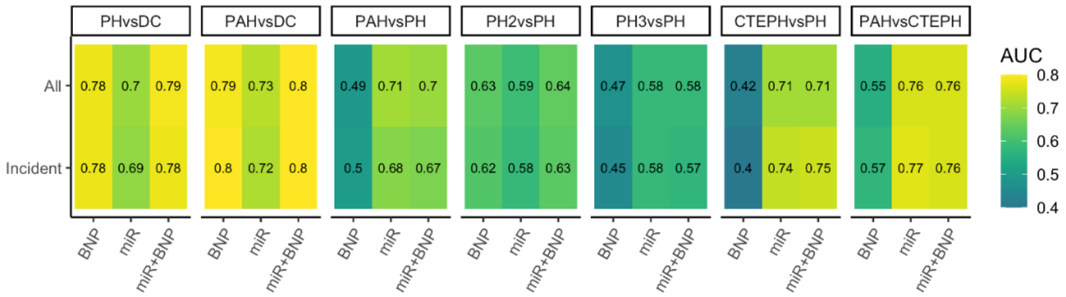

subgroup	PHvsDC	PAHvsDC	PAHvsPH	PH2vsPH	PH3vsPH	CTEPHvsPH	PAHvsCTEPH
All	251:96	97:96	97:154	41:210	34:217	74:177	97:74
Incident	200:95	64:95	64:136	41:159	33:167	58:142	64:58

B) Model performance in CIPHER cohort reveals no difference between treatment naive and all PH patients with each WSPH group.

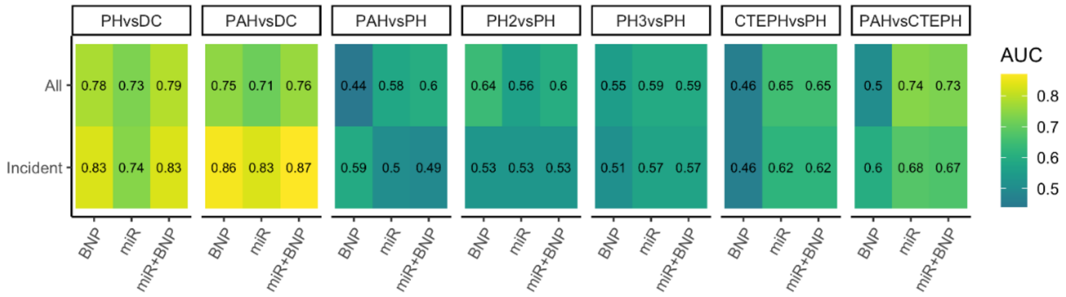

subgroup	PHvsDC	PAHvsDC	PAHvsPH	PH2vsPH	PH3vsPH	CTEPHvsPH	PAHvsCTEPH
All	349:93	135:93	135:214	82:267	29:320	68:281	135:68
Incident	164:69	53:69	53:111	39:125	18:146	31:133	53:31

Figure S5: miRNA signature model performance in incident and all patients within each WSPH group in the UK and CIPHER serum sub-study. miRNA model AUCs and incident patient compared to all patients for each comparison in the A) UK cohort and B) CIPHER study. Tables show patients numbers.
